# Effect of Surface Tooling Techniques of Medical Titanium Implants on Bacterial Biofilm Formation In Vitro

**DOI:** 10.3390/ma15093228

**Published:** 2022-04-29

**Authors:** Sonia Sarfraz, Pilvi-helinä Mäntynen, Marisa Laurila, Juho Suojanen, Juha Saarnio, Sami Rossi, Jani Horelli, Mika Kaakinen, Junnu Leikola, Justus Reunanen

**Affiliations:** 1 Biocenter Oulu & Cancer and Translational Medicine Research Unit, University of Oulu, 90014 Oulu, Finland; mika.kaakinen@oulu.fi; 2 Päijät-Häme Joint Authority for Health and Wellbeing, Lahti Central Hospital, Department of Oral and Maxillofacial Surgery, 15850 Lahti, Finland; pilvi-helina.mantynen@phhyky.fi (P.-h.M.); marisa.laurila@phhyky.fi (M.L.); juho.suojanen@helsinki.fi (J.S.); sami.rossi@fimnet.fi (S.R.); 3 Cleft Palate and Craniofacial Centre, Department of Plastic Surgery, Helsinki University Hospital, 00029 Helsinki, Finland; junnu.leikola@hus.fi; 4 Research Unit for Surgery, Anaesthesiology and Intensive Care, University of Oulu, 90014 Oulu, Finland; juha.saarnio@oulu.fi; 5 Planmeca, 00880 Helsinki, Finland; jani.horelli@planmeca.com; 6 Oulu Centre for Cell-Matrix Research, Faculty of Biochemistry and Molecular Medicine, University of Oulu, 90220 Oulu, Finland

**Keywords:** *Streptococcus mutans*, *Staphylococcus aureus*, *Enterococcus faecalis*, *Escherichia coli*, titanium, tooling, bacterial adhesion, saliva

## Abstract

The aim of this study was to assess the biofilm formation of *Streptococcus mutans*, *Staphylococcus aureus*, *Enterococcus faecalis*, and *Escherichia coli* on titanium implants with CAD-CAM tooling techniques. Twenty specimens of titanium were studied: Titanium grade 2 tooled with a Planmeca CAD-CAM milling device (*TiGrade 2*), Ti_6_Al_4_V grade 5 as it comes from CAD-DMLS device (computer aided design-direct metal laser sintering device) (*TiGrade 5*), Ti_6_Al_4_V grade 23 as it comes from a CAD-CAM milling device (*TiGrade 23*), and CAD-DMLS TiGrade 5 polished with an abrasive disc (*TiGrade 5 polished*). Bacterial adhesion on the implants was completed with and without saliva treatment to mimic both extraoral and intraoral surgical methods of implant placement. Five specimens/implant types were used in the bacterial adhesion experiments. Autoclaved implant specimens were placed in petri plates and immersed in saliva solution for 30 min at room temperature and then washed 3× with 1× PBS. Bacterial suspensions of each strain were made and added to the specimens after saliva treatment. Biofilm was allowed to form for 24 h at 37 °C and the adhered bacteria was calculated. Tooling techniques had an insignificant effect on the bacterial adhesion by all the bacterial strains studied. However, there was a significant difference in biofilm formation between the saliva-treated and non-saliva-treated implants. Saliva contamination enhanced *S. mutans*, *S. aureus*, and *E. faecalis* adhesion in all material types studied. *S. aureus* was found to be the most adherent strain in the saliva-treated group, whereas *E. coli* was the most adherent strain in the non-saliva-treated group. In conclusion, CAD-CAM tooling techniques have little effect on bacterial adhesion. Saliva coating enhances the biofilm formation; therefore, saliva contamination of the implant must be minimized during implant placement. Further extensive studies are needed to evaluate the effects of surface treatments of the titanium implant on soft tissue response and to prevent the factors causing implant infection and failure.

## 1. Introduction

The past few decades have seen explosive growth in the use of customized medical implants, especially in the field of cranio-maxillo-facial surgery, hand surgery, and hard tissue oncological surgery. Nowadays, various biomedical materials have been developed and utilized in clinical practice, but metal materials were the earliest developed, and are still the most extensively used materials in clinical implants. Titanium and its alloys are widely used in implant devices for their high resistance to corrosion, fatigue resistance, tensile strength, and, most importantly, good hard tissue compatibility. Conventional manufacturing processes do not fully provide biocompatibility to the medical implants; hence, surface treatments may be required to add biofunctions to the metals [1].

Implant and tissue interaction is crucial for successful implantation and it is frequently achieved with the modification of implant material before implantation [2]. One of the main goals of surgeons in orthopedics, maxillofacial, and dental surgery is to achieve excellent bone implant osseointegration. It has great clinical importance as it provides long-term stability to implants [3]. Implant surface characteristics greatly affect the process of osseointegration and the ultimate success of an implant treatment [4]. Several coordinated interactions occur between the implant material and the biological environment at their interface, and these events are affected by different surface topographies [5]. Milled implants are more often used in custom facial trauma surgery as well as in orthognathic surgery [6]. Laser sintering is used if larger pieces or three-dimensionally challenging structures are needed in implants, such as in reconstruction in surgical oncology [7,8]. There has been a study to evaluate the comparison of changes on titanium surface after treatment with two different types of ultrasonic tips. A conventional steel tip and an innovative copper alloy silver-plated tip were tested, and the results indicated that the novel copper alloy silver-plated one caused less damage to the titanium surface as compared to the old conventional steel tip. It is understood that irregularities on the surface of the implant promotes the growth of bacteria by enhancing the attachment of bacterial species on rough and irregular surfaces [9]. Microorganisms grow abundantly on the skin, and the soft and hard surfaces in the oral cavity, but with the advances in disease treatments and replacement procedures, the microorganisms have adapted to swamping the surfaces of the artificial implant materials as well [10]. 

Biofilms are clusters of bacterial cells of single or multiple species that are attached to each other and living or non-living surfaces, and they are encapsulated within extracellular polymeric matrices produced by them. These bacterial biofilms are responsible for chronic hospital-acquired infections. Of all bacterial infections, 65% are associated with biofilms [11]. There are some 700 bacterial species that inhabit the oral cavity, 400 out of which are responsible for biofilm formation on tooth and dental implants, which subsequently affect the tissues surrounding the implant [12]. It has been established that bacteria colonize peri implant interface soon after implantation to establish multi-microbial communities. Moreover, the biofilm formed on healthy implants is significantly different from biofilm formed on failing implants. In vitro studies have revealed that implant surface and composition also influence bacterial community structure in peri-implant spaces in implant dentistry [13]. In orthopedic implant infections, *Staphylococcus* and *Enterococcus* are the most frequently isolated microbes, whereas *E. coli* and other gram-negative bacilli are the least common microbes [14]. *Staphylococcus* accounts for approximately 65% of infections of prosthetic joint implantation [15] and is the most common pathogen in implant-related infections in neurosurgery, particularly in cranioplasty [16] and post-cochlear implant (CI) wound infections [17]. Similar results have been found in craniofacial surgery where alloplastic materials are exposed to pathogens due to impaired wound closure or wound dehiscence [18].

*Streptococcus* species are known as the earliest colonizers in the bacterial plaque. The role of early colonizers in the formation of biofilm is crucial as they provide foundation for the bacterial adhesion through growth and replication to the secondary colonizers [19,20]. *Streptococcus* spp. contributes as the second-most common microbe in prosthetic joint infections (PJI) caused by gram-positive cocci. It is responsible for 9–10% of PJI [21]. The incidence of orthopedic implant infections caused by gram-negative bacilli is low (10–23%) as compared to gram positive cocci. Among gram-negative bacilli, *E. coli* is the first and most common microbe to cause orthopedic implant infections [22]. There have been studies that detected the presence of *Enterobacteria* in superinfections related to periodontal diseases [23]. Enteric microorganisms such as *E.coli* are considered as non-residents in the oral cavity, but these transitory microbiota can act as opportunistic pathogens and have been identified subgingivally in patients with periodontal diseases [24,25]. The composition of microbes in peri-implantitis varies between different patients but representative bacteria found in most cases are *Streptococcus* and *Fusobacterium.* The frequent colonizers of dental implant infections in early and late implant losses are *Fusobacterium nucleatum* and *Porphyromonas gingivalis* followed by *Desulfobulbus* spp., *Pseudoramibacter alactolyticus*, *Treponema* spp., and *Fretibacterium* spp. [26]. Overall, *Staphylococcus* spp., *Streptococcus* spp., *Enterococcus* spp., and gram-negative bacilli among others are the main causes of biofilm infections associated with implants.

The aim of this study was to assess the biofilm formation of *S. mutans*, *S. aureus*, *E. faecalis,* and *E. coli* on titanium implants with different alloys and CAD-CAM tooling techniques, and to evaluate the effect of saliva on bacterial adhesion. Bacterial adhesions on the implants were completed with and without saliva treatment to mimic both extraoral and intraoral surgical routes for implant placement.

## 2. Materials and Methods

### 2.1. Titanium Implant Preparation

Titanium implants used in this study were from our CAD-CAM technical provider Planmeca Ltd. (Helsinki, Finland). All 20 implants were manufactured as 20 mm discs, destained and sterilized in a similar manner to that normally provided for the needs of patient-specific cranio-maxillo-facial surgery in clinical practice [6,7,8,27]. Implant types studied were: Titanium grade 2 tooled with a Planmeca CAD-CAM milling device (TiGrade 2), Ti_6_Al_4_V grade 5 as it comes from a CAD-DMLS device (computer aided design—direct metal laser sintering device) (TiGrade 5), Ti_6_Al_4_V grade 23 as it comes from a CAD-CAM milling device (TiGrade 23), and CAD-DMLS TiGrade 5 polished with an abrasive disc (TiGrade 5 polished). Disc samples “TiGrade23” and “TiGrade2” were manufactured with a CORiTEC Imes icore 450i milling machine. These discs’ surfaces were not finished or polished in any way. Surfaces were left as they were after milling. Disc samples “TiGrade5” and “TiGrade5 polished” were manufactured with an EOS M 280 metal printer (DMLS). Both sample series went through the common finishing process (for DMLS 3D metal additive manufacturing) which includes heat treatment, grinding, sand blasting, and passivation. The “TiGrade5 polished” sample series was additionally polished with abrasive disc (Orbis rubber pin, MD 125348, Munster, Germany, Orbis dental, Munster, Germany) without any additional paste. All sample series were cleaned ultrasonically before sterilization [6,7,8].

### 2.2. Saliva Contamination

Sterile saliva was used for saliva treatment. Saliva was collected from healthy volunteers using paraffin wax stimulation. Stimulated saliva was pooled and filtered using a 0.45 µm filter (#167-0045 Nalgene™ Rapid-Flow™ Sterile Single Use Vacuum Filter Units, Nalgene^®^ 295-4545, Edo, de México, Mexico). The filtered saliva was stored at −80 °C. Before usage, saliva was diluted 1:1 in 1× phosphate buffer saline (PBS). Before the addition of the buffer, the pH of the saliva was measured at 7.6, and after addition of the buffer it was 7.5, which is within the range of normal salivary pH (6.2–7.6). Autoclaved titanium specimens were first immersed in saliva solution for 30 min at room temperature followed by 3× washes with 1× PBS.

### 2.3. Bacterial Strains and Suspension Preparation

*S. aureus* (SM) DSM 29134, *S. mutans* DSM 20523, and *E. faecalis* DSM 20380 were bought from the Leibniz Institute DSMZ-German Collection of Microorganisms and Cell Culture GmbH, and *E. coli* was isolated from a human fecal sample. The first three bacterial strains were cultured in a trypticase soy yeast extract medium and *E. coli* was cultured in LB (Lysogeny broth).

The bacterial adhesion of each strain was completed following the same protocol. Overnight bacterial culture was taken and centrifuged for 10 min at 8000× *g* to pellet the bacteria. Bacterial pellets were suspended in 1× PBS and centrifuged again for 10 min at 8000× *g*. Washed bacterial pellets were then diluted to OD600 = 0.25 with the respective growth media.

### 2.4. Biofilm Formation

Five specimens/groups were used in the bacterial adhesion experiments. Autoclaved specimens with or without saliva contamination were placed in petri plates and bacterial cultures were added. Then, the plates were sealed with parafilm and incubated for 24 h at 37 °C.

### 2.5. Enumeration of Adhered Bacteria

Culture media containing non-adherent bacteria was removed and specimens were carefully washed three times with 1× PBS. The specimens were then transferred to six-well plates, with each well containing one specimen. A total of 1 mL of 1× PBS was added to each well containing a specimen. Biofilm was scraped using dental brush sticks (EAN 7630019902762) and their tips were shaken repeatedly in 1× PBS to ensure detachment of bacterial cells from the brushes followed by the collection of 1× PBS in Eppendorf tubes. Serial dilutions were made to enumerate the adhered bacteria. Trypticase soy yeast extract medium agar and LB agar plates were used for the respective strains. For CFU (colony-forming unit) counting, agar plates were incubated for 48 h at 37 °C.

### 2.6. Statistical Analysis

*p* values were determined using *t*-Test: Two-Sample Assuming Unequal Variances. *p* values < 0.05 were considered statistically significant.

### 2.7. Treatment of Samples for Scanning Electron Microscopy (SEM)

Bacterial biofilm was grown for 24 h, followed by the removal of non-adherent bacteria and the washing of specimens with 1× PBS. The specimens containing attached biofilm were fixed with 1% glutaraldehyde, 4% paraformaldehyde in 0.1 M phosphate buffer, air dried, and then sputter coated with a 5 nm platinum layer. Imaging was recorded with the Sigma HD VP FE-SEM by using an In-Lens detector.

## 3. Results

### 3.1. Bacterial Adhesion and Biofilm Formation

**Figure 1:**
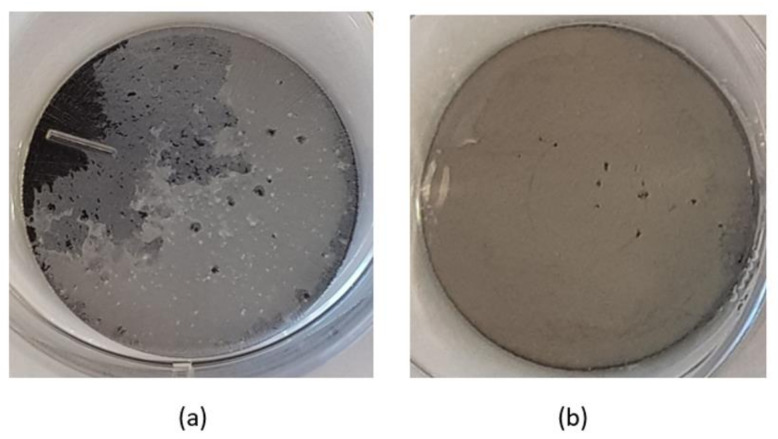
Bacterial biofilm formation on different titanium discs (**a**) *S. mutans* biofilm on Ti grade 23, (**b**) *S. aureus* biofilm on Ti grade 5 polished.

Representative images of titanium discs showing bacterial biofilm formation are presented in Figure 1. These images were taken after the washing of loosely attached bacterial biofilm with a PBS buffer.

**Figure 2:**
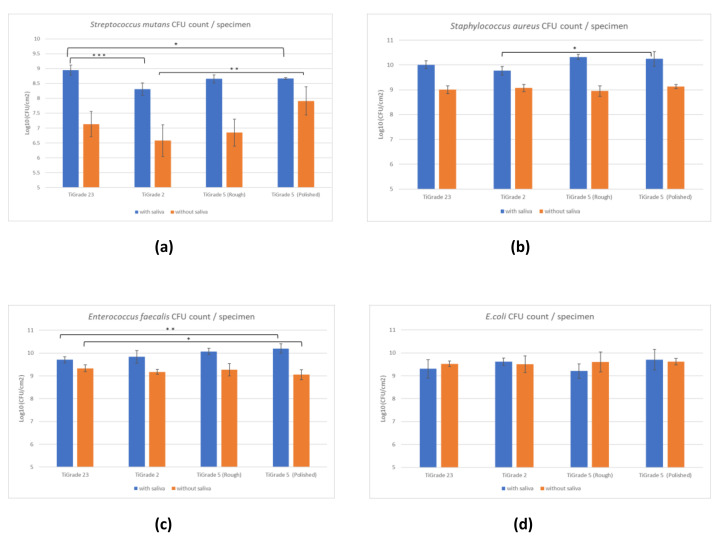
Comparison of colony-forming unit (CFU) count of (**a**) *S. mutans*, (**b**) *S. aureus*, (**c**) *E. faecalis* and (**d**) *E. coli* on different titanium implant materials: with saliva treatment (blue) and without saliva treatment (orange). Statistical difference between materials is marked with lines * *p* < 0.05, ** *p* < 0.01, *** *p* < 0.001.

The results of bacterial biofilm formation experiments are presented in Figure 2. Tooling and milling techniques slightly affected bacterial adhesion. However, there was a significant difference in bacterial adhesion between the saliva-treated (S) and non-saliva-treated (NS) groups.

*S. mutans* adhesion in the S group was significantly greater in Ti grade 23 as compared with Ti grade 2 (*p* = 0.0007) and Ti grade 5 polished (*p* = 0.02). In the NS group, *Streptococcus* adhesion was significantly larger in Ti grade 5 polished than Ti grade 5 (*p* = 0.006), Ti grade 2 (*p* = 0.003), and Ti grade 23 (*p* = 0.02).

For the S group, *S. aureus* adhesion was found to be significantly higher in Ti grade 5 polished than Ti grade 2 (*p* = 0.02). However, there was no difference in *S. aureus* adhesion in the NS group (*p* = 0.1). In the S group, *E. faecalis* showed more attachment in Ti grade 5 polished than Ti grade 23 (*p* = 0.002). In the NS group, Ti grade 23 had larger binding than Ti grade 5 polished (*p* = 0.04). There was no significant difference in adhesion between other materials (*p* = 0.2) in both the S and NS groups. *E. coli* showed no significant difference in biofilm formation between materials in both the S (*p* = 0.08) and NS (*p* = 0.5) groups.

In comparison, *Streptococcus*, *Staphylococcus* and *Enterococcus* adherence was found to be greater in the S group than in the NS group. For *S. mutans*, the increase in biofilm formation induced by saliva was more profound than with other bacterial strains.

Overall, *S. aureus* had the highest attachment in the S group, whereas *E. coli* showed the largest biofilm formation in the NS group. The most significant difference between materials was found in Ti grade 2, as it showed the least bacterial adhesion by *S. mutans* in both the saliva-treated and non-saliva-treated groups.

### 3.2. SEM Analysis of Biofilms

**Figure 3:**
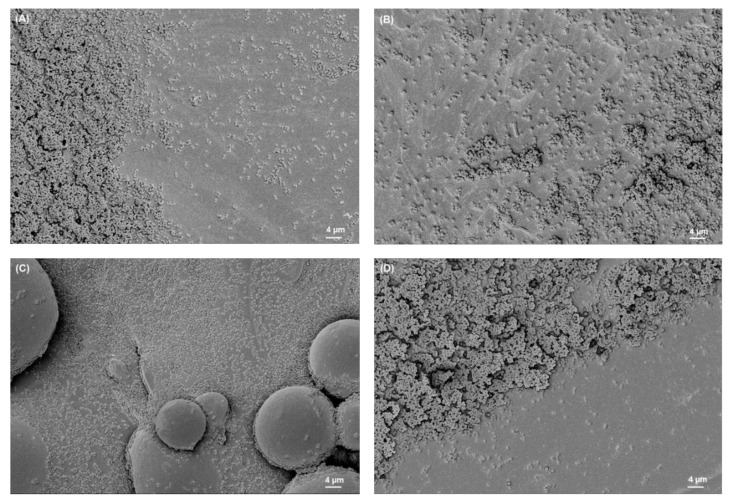
*S. aureus* biofilm on (**A**) Ti grade 23, (**B**) Ti grade 2, (**C**) Ti grade 5, (**D**) Ti grade 5 polished. All images were recorded at high voltage of 5 kV, magnification = 1.18 KX with scale bars = 4 µm.

**Figure 4:**
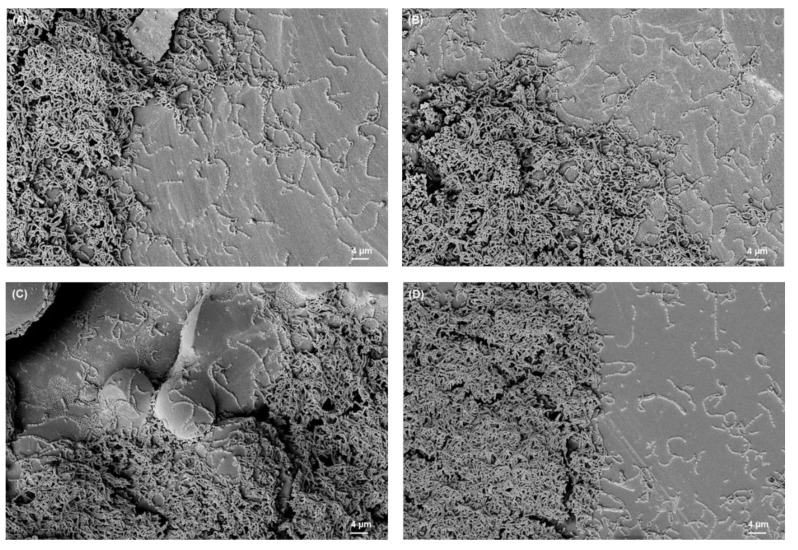
*S. mutans* biofilm on (**A**) Ti grade 23, (**B**) Ti grade 2, (**C**) Ti grade 5, (**D**) Ti grade 5 polished. All images were recorded at high voltage of 5 kV, magnification = 1.18 KX with scale bars = 4 µm.

**Figure 5:**
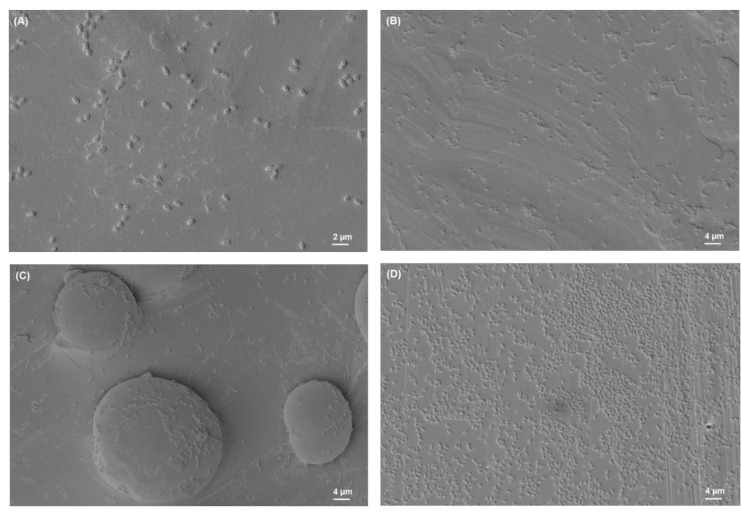
*E. faecalis* biofilm on (**A**) Ti grade 23, (**B**) Ti grade 2, (**C**) Ti grade 5, (**D**) Ti grade 5 polished. All images were recorded at high voltage of 5 kV, magnification = 1.18 KX with scale bars = 4 µm (except image (A), magnification = 2.22 KX, scale bar = 2 µm).

**Figure 6:**
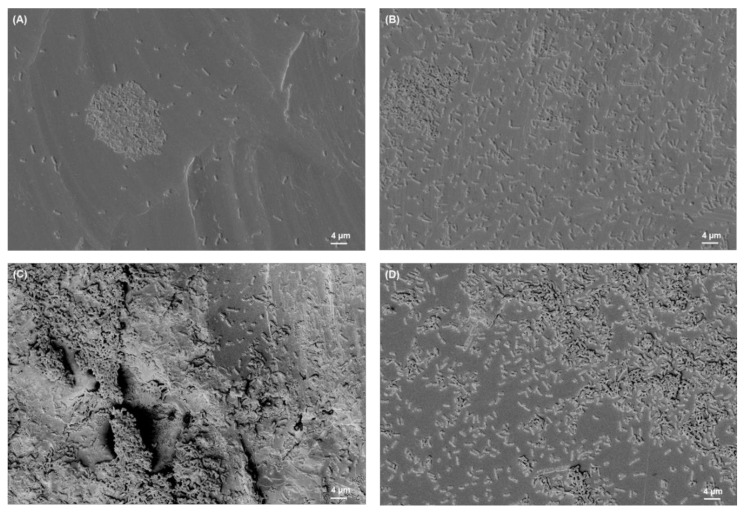
*E. coli* biofilm on (**A**) Ti grade 23, (**B**) Ti grade 2, (**C**) Ti grade 5, (**D**) Ti grade 5 polished. All images were recorded at high voltage of 5 kV, magnification = 1.18 KX with scale bars = 4 µm.

The SEM images of biofilms on different titanium implant materials are shown in Figure 3, Figure 4, Figure 5 and Figure 6. These images were obtained after the washing of loosely attached biofilms.

## 4. Discussion

The aim of this study was to assess the biofilm formation of common surgical infection potential-exerting bacteria on CAD-CAM surgical titanium implants. The effects of alloy selection and CAD-CAM tooling techniques were studied with materials widely used in maxillofacial and hand surgery. In vitro studies with the implants were designed to be performed with and without salivary coating to mimic both extraoral and intraoral surgical approaches to implant placement. Salivary coating seemed to enhance the adhesion of *S. mutans*, *S. aureus* and *E. faecalis* on all tooling and alloy types studied. Tooling techniques (milling, laser sintering, and polishing) had a considerably smaller effect on bacterial adhesion. This is an interesting finding since macroscopic difference on material roughness is rather notable between native laser sintering and polished material. In the present study, polished titanium (Ti6A14V grade 5) in general showed higher bacterial adhesion as compared to other alloy types, but this difference is not statistically significant. These results are in accordance with the study which showed an increased adherence of periodontopathic bacteria on polished titanium surfaces [28]. Similar studies conducted to access the bacterial adhesion on different titanium implant materials showed varied results [29,30]. These findings indicate that surface treatments could be beneficial in the prevention of the initial colonization of implant surfaces.

In the current study, *S. mutans* was found to be the least-adhered bacteria out of all strains tested. A similar result was concluded in a study to investigate the adhesion of *Streptococcus* to titanium and ceramic implant surfaces, which demonstrated that the potential of *Streptococcus* to adhere to other implant materials was significantly higher than that to titanium [31]. In a study carried out to evaluate the adherence of *Streptococcus* to different implant materials after salivary coating, it was indicated that the extent of bacterial adhesion also depends on the nature of the bacterial strains and the substrata [32].

Of the bacteria studied, *S. aureus* was the most adherent species. Interestingly, salivary treatment notably enhanced its adhesion. This may be an important finding for designing surgical access for medical implantation or osteosynthesis; it may be wise to avoid simultaneous extraoral and intraoral approaches where both the salivary and the cutaneous contamination of a surgical implant are possible. Additionally, special patient groups, such as cleft lip and palate patients with oro-nasal fistulas can be more prone to *Staphylococcus* surgical implant infections since *S. aureus* commonly colonizes the nasal cavity [33].

In a 10-year retrospective study, 1% of 1247 titanium miniplates used in maxillofacial surgery were removed for the reason of a superficial infection; this number represented 41% of all plates removed, as the total removal rate was 3% [34]. Patient-specific implants (PSI) can be completely manufactured out of titanium, significantly diminishing the risk of an allergic reaction and implant failure compared to stock implants containing Co-Cr-Mo to which 10% of the population is allergic [35]. With titanium alloys the cell and tissue response are improved, resulting in very few adverse tissue reactions, and close apposition between the implant and bone is established [36,37]. However, titanium can also cause an allergic reaction. To establish a rigid connection between the implant and the bone (osseointegration), direct bone anchorage is needed without the formation of an intervening fibrous tissue layer [38]. Porosity enables bone ingrowth but can also result in soft tissue entrapment, as 3D printing cannot currently create smooth upper surfaces and porous under surfaces [39]. Mommaerts et al. have discussed in their paper that a porous structure consisting of diamond unit cells enables bony ingrowth, but conversely, porosity hampered the production and impaired accuracy of the PSI when bone contact was limited, also causing a higher infection risk [40]. However, our current adhesion data suggest that implant surface structure and roughness is favorable to design based on soft tissue response and osseointegration rather than the possible risk of a rough surface on bacterial infections.

As discussed in a study on biomaterials in temporomandibular joint replacement, [38] the surface chemistry of the implant, as well as the topography and surface energy, is an important factor in the implant-tissue interaction at the implant surface, starting from protein absorption to cellular adhesion, proliferation, and differentiation to tissue development [38]. A higher surface energy i.e., hydrophilicity, is more favorable for cell attachment [41]. Then again, a titanium surface modified by small silane molecules containing hydrophobic alkyl chain and a positively charged functional group can effectively counter bacterial adhesion and growth. After 30 min of incubation, 91.7% of the initial adherent reduction against *S. aureus*, 91.3% against *P. aeruginosa,* and 90% against *E. coli* was found [42]. A core temperature <34.7 °C (1.4 °C below normal) during general anesthesia may increase wound infection [43]. Patients need to be externally warmed because hypothermia can cause wound infection as well as other disadvantages [44]. Vasoconstriction decreases the subcutaneous oxygen tension and impairs T-cell-mediated antibody production and bacterial killing by neutrophils [45]. In intraoral incisions, infection control is maintained by copious wound rinsing, rinsing the implant in antibacterial solution, double-layer wound closure, the application of fibrin glue under the suture line, and with prophylactic antibiotics perorally continued for five days post-operatively. TiO_2_ nanostructures, used as reinforcement materials or coatings for the bare surface of titanium implants, can compensate titanium implant deficiencies such as poor surface interaction with surrounding tissues, by providing nanoporous surfaces and hierarchical structures. These nanostructures can also be loaded by diversified drugs such as antibiotics. Toxicity and biocompatibility of TiO_2_ nanostructures need to be considered [46]. The attachment of implant coatings has been problematic in screwed titanium dental implants, and whether they could prevent bacterial infections in CAD-CAM surgical implants remains to be investigated. 

In recent years there has been a lot of debate about the toxicity and safety of titanium and its alloys. Grade 5 titanium alloy Ti6Al4V is successfully widely used in dental and medical implants. There is some evidence of the toxicity of vanadium in medical devices, hence orthopedics have stopped the use of vanadium-containing implant alloys. In contrast, Ti6Al4V is still widely used in orthodontics and oral and maxillofacial surgery. There is evidence that dental implants also release cytotoxic vanadium and aluminum wear and corrosion particles to surrounding tissues in vitro compared to pure titanium; this finding should be further investigated in clinical settings [47,48]. Titanium implant surfaces can be further modified with nanoscale ultrafine nanoparticles to enhance implant biocompatibility and antimicrobial activity. Antimicrobial Ag, ZnO, CuO, and Quercitrin nanoparticle coatings have been studied with success. Coatings with Al_2_O_3_, Hydroxyapatite, Calcium phosphate, TiO_2_ and nano-crystalline diamond have been used successfully to enhance osseointegration. However, good in vitro results of nanoparticle coatings still need to be further investigated in clinical setting before commercial use [49]. Additionally, the surface energy and crystalline phase of modified TiO_2_ implant surface nanoparticles play a role in the immunogenic response of the surrounding tissues; there is evidence that an anatase crystalline phase can cause local inflammation [50]. These findings are significant as implant surface nanoparticles can cause adverse effects on surrounding tissues and even general health.

Various studies have been completed to evaluate the tools for the analysis of implant and bone morphology. The regeneration or repair of bone is commonly investigated through X-ray and computed microtomography (µCT) along with histological examination of samples. On the other hand, magnetic resonance imaging (µMRI) gives a more comprehensive analysis of tissue as compared to CT. A study was conducted to develop a multimodal 3D imaging technique based on the µCT and µMRI techniques combined in order to provide a non-destructive way of examining bone. The aim of this study was to connect the gap between high quality 2D imaging and 3D microtomographic techniques using a multimodal approach and they were able to successfully differentiate and examine the newly-formed bone from the old, calcified bone [51]. Another study reviewed the advantages of MRI in endodontics and suggested that MRI is more suitable to visualize soft tissue as it not only diagnoses soft tissue diseases without using ionizing radiation, but also differentiates vessels and nerves [52].

There are some limitations to the present study, as it evaluated the bacterial biofilm formation in vitro. It is important to realize that the in vitro environmental setting is relatively different from conditions in the human body. The proteins and immune response that occur in the human body are missing in in vitro conditions. Filtration of saliva causes not only a considerable decrease in the total protein concentration but also a selective loss of certain proteins such as lysozymem which plays an integral part in the host immune response [53]. Additionally, in clinical conditions, there are several bacterial species involved at the same time which could affect bacterial adhesion. We tried to eliminate these variables by selecting the bacterial species that are most commonly found in implant-related infections and by using saliva treatments to mimic the oral cavity. The sample size was chosen according to previous similar studies [9,54], and the results obtained with this sample size in the present study showed statistically significant differences (*p* < 0.001) between materials in some cases. 

## 5. Conclusions

These conclusions are drawn within the limitations of the present in vitro study. The CAD-CAM technique or the polishing of the titanium implant have rather little effect on bacterial adhesion and biofilm formation. Saliva enhances bacterial adhesion and for this reason contamination of the implant with saliva must be minimized when performing surgery. Surface roughness can potentially affect soft tissue adherence on implant surfaces, and this may possibly have a significant effect on wound dehiscence, which can also lead to implant infection. Therefore, future in vitro assays with fibroblast adhesion as well as in vivo models and possibly clinical studies are warranted to evaluate soft tissue response and wound closure effects on bacterial adhesion. 

## Data Availability

Not applicable.
